# Is genicular nerve radio frequency ablation the key to improving patients’ satisfaction after total knee arthroplasty? a randomised controlled trial

**DOI:** 10.1186/s13018-026-06855-8

**Published:** 2026-04-25

**Authors:** Assem M. M. Ahmed, Ahmed Sayed Kotb, Amr Khairy, Ibrahim El Ganzoury, Haytham Abdelazim, Ahmed Mohamed Mohasseb

**Affiliations:** 1 Faculty of Medicine, Ain Shams University, Cairo, Egypt; 2 Faculty of Medicine, Ain Shams University, Cairo, Egypt; 3 Faculty of Medicine, Ain Shams University, Cairo, Egypt; 4 Department of Orthopedic Surgery, Faculty of Medicine, Ain Shams University, Ramses St., Abbasiya sq, Cairo, 11566 Egypt

**Keywords:** Genicular nerve radio-frequency ablation, Total knee arthroplasty, Postoperative pain, Randomized controlled trial

## Abstract

**Background:**

Some studies regarding patients complaining of residual pain after total knee arthroplasty (TKA) showed pain scores improvement using genicular nerve radiofrequency ablation (GNRFA). This prompted the hypothesis that combining GNRFA with TKA intraoperatively might improve early postoperative pain control and functional outcomes.

**Methods:**

Seventy patients were randomly assigned into two groups, one group underwent total knee arthroplasty combined with genicular nerve radiofrequency ablation (TKA-GNRFA), and the other group underwent total knee arthroplasty (TKA) alone. A parallel-group trial with 1:1 allocation using a superiority framework, Visual Analog Scale (VAS), and functional outcome using the Oxford Knee Score (OKS) were evaluated. Assessments were conducted during the first six months following surgery.

**Results:**

There were no significant differences in pain score or functional outcome between the two groups in the first six months postoperative follow-up period.

**Conclusion:**

At Six months postoperatively, Combining Genicular Nerve radiofrequency ablation with total knee arthroplasty (TKA-GNRFA) showed no significant advantage regarding pain scores and functional outcome over performing TKA alone. Further randomized controlled trials with larger sample sizes are recommended to provide higher-level evidence and validate these findings.

*Trial Registration* Retrospectively registered under Trial Registration Number NCT07381062 on 24-01-2026.

## Introduction

Total knee arthroplasty is a very common operation, and its incidence is believed to increase further in the near future [1]. That’s why there are a lot of studies focused on improving the patient’s satisfaction rate, while the unsatisfied patients remain varying from 10–30% [2]. Early postoperative rehabilitation and fast track protocols showed improvement in functional outcome and pain relief but are still hindered by the patient’s compliance [3]. Our aim to improve TKA satisfaction led to the idea of implementing genicular nerve radio-frequency ablation in combination with TKA. The hypothesis was developed after when GNRFA showed significant improvement in patients with chronic knee osteoarthritis. Studies showed pain relief up to 67% in the first three months, yet alone it has limited indications as it addresses only the biological aspect of the knee osteoarthritis without resolving the mechanical problem that will continue to progress over time [4]. Other studies showed positive outcome of GNRFA in cases of chronic knee pain after TKA [5, 6].

## Patients and methods

A randomized controlled clinical trial was conducted in Ain Shams University Hospitals including 70 knees Participants were randomly assigned to either the TKA-GNRFA or TKA-only group in a 1:1 ratio using a computer-generated random sequence. To ensure allocation concealment, the sequence was maintained by an independent researcher not involved in participant recruitment or surgical procedures. Group assignment for each patient was revealed to the surgical team via sequentially numbered, opaque, sealed envelopes only after the patient was in the operating room and prepared for surgery.

This was a single-blinded trial. Due to the nature of the surgical intervention, it was not possible to blind the operating surgeon. However, participants remained blinded to their group allocation throughout the six month follow-up period. Additionally, the outcome assessors responsible for recording the Visual Analog Scale (VAS) and Oxford Knee Score (OKS) data, as well as the data analyst, were kept unaware of group assignments until the final statistical analysis was complete.

All TKA surgeries were performed by a single surgeon including patients aged between 50 and 80 years with advanced primary knee osteoarthritis from both sexes. We excluded patients who underwent previous knee surgery or prior GNRFA in the same knee and patients requiring constrained knee arthroplasty prosthesis (with extreme deformities). We had no changes to the trial protocol after commencing.

*Sample Size*: By Using PASS 11 program for sample size calculations “NCSS 2022 Statistical Software (2022). NCSS, LLC. Kaysville, Utah, USA, ncss.com/software/ncss.” Setting the power at 99% alpha error at 0.05, Minimal Clinically Important Difference (MCID) of 1.7 points for the Visual Analog Scale (VAS) and 4.3 points for the Oxford Knee Score (OKS). Using a standard deviation of 1.5 for VAS and 3.8 for OKS sample size calculated at least 60 knees, to account for potential dropouts, 70 knees were enrolled (35 per group).

Ethical Considerations: This study was performed in accordance with the Declaration of Helsinki. We obtained Faculty of Medicine, Ain Shams University Research Ethics Committee approval with reference number FMASU MD39/2022.

This study was Retrospectively registered on clinicaltrials.gov under Trial Registration Number NCT07381062 on 24-01-2026, https://clinicaltrials.gov/study/NCT07381062?term=NA.

### Preoperative

Diagnosis and evaluation were done by history taking, general examination and local examination. Primary outcomes, VAS and OKS were assessed. Radiological evaluation was done using plain x-ray standing antero-posterior, lateral and sunrise view for the diseased knee. Routine pre-operative laboratory investigations were done for all patients.

## Operative technique

In the (TKA-GNRFA) group the GNRFA was done first using Neurotherm^®^ NT2000iX Wilmington, United States. The patients were positioned supine on the operating table, grounding pad was placed on the contralateral thigh and connected to the Radiofrequency (RF) generator, the diseased knee was placed in a 30° of flexion and draped in a sterile manner. The participants were provided with conscious sedation before starting the procedure, then using fluoroscopy three anatomical zones are identified [7].

The superior lateral genicular nerve is located at the junction of the lateral femoral shaft and the femoral condyle in the anteroposterior plane and midpoint of the femur in the lateral plane.

The superior medial genicular nerve is located at the junction of the medial femoral shaft and the femoral condyle in the anteroposterior plane and midpoint of the femur in the lateral plane.

The inferior medial genicular nerve is located at the junction of the medial tibial shaft and the tibial flare in the anteroposterior plane and midpoint of the tibia in the lateral plane [8].

The superior medial quadrant is meant to affect the nerve to vastus medialis, nerve to vastus intermedius and the superior medial genicular nerve, the superior lateral quadrant is meant to affect the nerve to vastus lateralis, superior lateral genicular nerve and the articular branch of the common fibular nerve, the inferior medial quadrant is meant to affect the inferior medial genicular nerve and the infrapatellar branch of the saphenous nerve as shown in the anatomical study done by Tran et al. [9].

A 2 ml Lidocaine injection was given to form a skin wheel prior to the insertion of the percutaneous cannulas in the three aforementioned sites. With the assistance of the fluoroscopy the cannula was inserted in the targeted locations then the radiofrequency probe is inserted inside the cannula to perform two tests.

Sensory stimulation was done using the parameters of 50 Hz confirming the proper positioning of the cannula, usually the patient was conscious enough to complain of knee pain or pressure similar to the pain he/she usually experiences. And this step can be skipped in some of the literature as the main confirmation is the fluoroscopic anatomical site.

Motor stimulation was done using the parameters of 2 V 2 Hz to assure that there is no undesired motor nerves affected which will be confirmed if no movement or fasciculations occur.

Then after both tests were confirmed, we injected 2 ml of lidocaine in the cannula and waited for 90 s to have the full effect. After that we proceeded with the nerve ablation by re-inserting the radiofrequency electrode and started at the temperature of 80° for 2 min. Lastly, we removed the electrode and injected 1 ml of methylprednisolone to prevent the possibility of neuritis and then removed the cannulas and proceeded with the TKA [10].

Total knee arthroplasty was done using the same technique in both groups. Spinal anesthesia was used for all patients. one gram of tranexamic acid and 1 gram of cephalosporin was given 30 min to 1 h before skin incision, torniquet was used during the whole operation and deflated before closure for hemostasis.

A standard mid-vastus approach with measured resection TKA technique using Max Orthopedics Freedom^®^ Knee, Norristown, United States and patellar denervation was done for all the patients. After the application of the prothesis the torniquet was deflated and another 0.5gm of tranexamic acid was administered, adequate hemostasis was done and a prepared cocktail of 20 ml ropivacaine, 0.5gm of tranexamic acid and 1gm of cephalosporin is added up with saline to 60 ml and injected in the whole medial aspect (quadriceps and patellar tendons as well as subcutaneous), then a drainage tube is inserted prior to wound closure [11].

## Postoperative

After the surgery all patients were connected to a patient-controlled intravenous analgesia (PCIA) pump. Postoperative anteroposterior and lateral knee radiographs were obtained. Weight-bearing was allowed on the first postoperative day, and drains were removed within 24 h. Standard DVT prophylaxis, antibiotics, NSAIDs, and paracetamol were prescribed.

## Follow up

Patients were assessed for pain using the VAS on follow up periods 2,6,12 and 24 weeks, and for functional outcome using OKS on 6,12 and 24 weeks.

## Results

Trial recruited participants between February 22, 2022, and March 4, 2024. A total of 82 patients were screened for eligibility. Of these, 12 were excluded: 7 did not meet the inclusion criteria; requiring constrained prosthesis and 5 were eligible and not approached.

Following the screening, 70 participants were successfully randomized: 35 to the TKA-GNRFA group and 35 to the TKA-alone group. All participants in both groups received their allocated surgical intervention as planned.

During the six-month follow-up period, one patient was lost to follow-up in the TKA-GNRFA group, and 1 was lost in the TKA-alone group for changing contact information. A Per-Protocol analysis was conducted, including only those participants who completed the full six-month follow-up and received the assigned intervention without protocol deviations. Missing data for the primary and secondary outcomes were handled using the Last Observation Carried Forward (LOCF) method.

The collected data was revised, coded, tabulated and introduced to a PC using Statistical package for Social Science (SPSS 27). Data was presented and suitable analysis was done according to the type of data obtained for each parameter.

The mean age group in the study is 63.1 years, and the mean body mass index (BMI) is 33.2. The study population had higher female ratio with 73.5% of the patients being females. Hypertension (HTN) was the most common comorbidity with 64% of the patients having hypertension, followed by Diabetes mellitus (DM) with 29% of the patients. No difference in the osteoarthritis grading was noted between the two groups with the majority being Kellgren-Lawrence grade 4. There were no statistically significant differences in the sociodemographic data between the 2 study groups.

Preoperative median VAS scores were slightly higher in TKA-GNRFA than the TKA group with 6 and 5 respectively. TKA-GNRFA had slightly lower VAS six weeks post-operative with a median of 2 than the TKA group with a median of 3, yet the difference in the scores were statistically insignificant, Table 1.

**Table 1:** VAS assessment between two study groups.

	Group		Mann-Whitney test		
	Total Knee arthroplasty	Total Knee arthroplasty + Genicular nerve radiofrequency ablation			
	Median (IQR)	Median (IQR)	z	*P*-value	Sig.
Pre	5 (5–6)	6 (5–6)	−0.389	0.697	NS
After 2 weeks	3 (3–3)	3 (3–3)	−0.578	0.563	NS
After 6 weeks	3 (2–3)	2 (2–3)	−1.898	0.058	NS
After 3 months	1 (1–2)	1 (1–2)	−0.120	0.905	NS
After 6 months	1 (0–1)	1 (0–1)	−0.214	0.831	NS
Pairwise comparisons (all pairs)	< 0.001*	< 0.001			

^*^All pairs *P*-value were (< 0.001), except 2 weeks versus 6 weeks it was (0.037)

Pairwise comparisons were done using Wilcoxon signed rank test of significance

The OKS showed very close mean results with gradual improvement in both groups, Table 2.

**Table 2:** OKS assessment between two study groups.

	Group		Student t-test		
	Total knee arthroplasty	Total knee arthroplasty + Genicular nerve radiofrequency ablation			
	Mean ± SD	Mean ± SD	t	P-value	Sig.
Pre	16.71 ± 4.45	17 ± 4.26	−0.278	0.782	NS
After 6 weeks	28.88 ± 3.88	28.94 ± 4.04	−0.061	0.951	NS
After 3 months	35.24 ± 4.07	35.41 ± 3.92	−0.182	0.856	NS
After 6 months	38.44 ± 3.15	38.41 ± 3.47	0.037	0.971	NS
Pairwise comparisons (all pairs)	< 0.001	< 0.001			

Pairwise comparisons were done using Paired t-test of significance

One patient in each group reported as unsatisfied. In the GNRFA-TKA we had 2 complications with the first one being a calf ulcer resulting from the elastic bandage wrapped around his limb and the other one was an infection that underwent debridement, antibiotics, irrigation and implant retention (DAIR) with exchange of the plastic insert and the infection resolved. In the TKA group there was one patient who suffered from prolonged serous discharge that was resolved spontaneously, and another patient that suffered from post-operative infection and was resolved by DAIR, we didn’t encounter any GNRFA specific complications such as site hematoma, neuritis or motor nerve affections, Table 3.

**Table 3:** Post-operative data between two study groups

		Group		Fisher’s exact test	
		Total knee arthroplasty	Total knee arthroplasty + Genicular nerve radiofrequency ablation		
		N (%)	N (%)	*P*-value	Sig.
Satisfaction	No	1 (2.94%)	1 (2.94%)	1.00	NS
	Yes	33 (97.06%)	33 (97.06%)		
Complications	No	32 (94.12%)	31 (91.18%)	1.00	NS
	Calf ulcer resolved with dressing	0 (0%)	1 (2.94%)		
	Delayed wound closure (Serous discharge)	1 (2.94%)	0 (0%)		
	Infected resolved with debridement	1 (2.94%)	1 (2.94%)		

This table shows 20.59% of the TKA group still suffered from residual pain and 14.71% in the TKA-GNRFA group 6 months post-operative, Table 4.

**Table 4:** Residual pain at six months

		Group				Chi square test		
		Total knee arthroplasty		Total knee arthroplasty + Genicular nerve radiofrequency ablation				
		Count	Column N %	Count	Column N %	Value	*P* value	Sig.
6 m	No pain	27	79.41%	29	85.29%	0.405	0.525	NS
	Residual pain	7	20.59%	5	14.71%			

## Discussion

This randomized controlled clinical trial was conducted to assess the effect of adding GNRFA to TKA on Postoperative pain, functional outcome and patients’ satisfaction. Most of the studies refer to an unsatisfactory TKA operation as a multifactorial problem, which leads to very difficult approaches, and most of them still recommend further investigation into the problem. As a lot of studies related to the unsatisfaction for multiple reasons, three main reasons are addressed the most being, persistent pain, functional impairment, and unmet pre-operative expectations [12–16]. Our study hypothesized that implementing a recent pain management technique that has been used in some studies as a management for painful TKA as a routine approach, part of a multi-disciplinary protocol could help improve the pain and functionality, hence the overall satisfaction of the procedure. The GNRFA was recently introduced as a possible management for painful total knee in some studies with good outcome [17–20].

There were no statistically significant variations in pre-operative demographics between the two groups, the two primary outcomes were pain assessed by the VAS and the functional outcome represented by OKS which is well suited scoring system to assess the functionality after TKA, both these scores were assessed pre-operatively to account for possible variations between the two groups but there were no significant difference, the improvement in the scoring systems were not accounted for in our study since the primary objective is measuring the effectiveness of GNRFA when performed with TKA in comparison to a control group. Yet the VAS and OKS reported in our study were comparable to previous studies [21–24].

There were two acute periprosthetic joint infection (PJI) complications reported in our study representing 2.9% which is comparable to the reported rate in literature 25. The two infected knees were managed according to the modified Musculoskeletal Infection Society (MSIS) criteria for periprosthetic joint infection (PJI) published in 2018 [26], both cases underwent DAIR within the first 10 days of the operation and the infection was eradicated successfully. The complication rate in both groups didn’t signify any increase in complication rate in either of the study groups.

The satisfaction rate in our study was assessed as a simple yes or no question, on whether the patient is satisfied with the whole experience or not. Using this tool 97.06% of the patients reported satisfied, referring to the major improvement they experienced within the 6 months period after the operation, yet this doesn’t mean that all these patients were 100% satisfied correlating this to the 6 months pain scores whether there is any residual pain or not. In the satisfied patients own words it was always “I am way better than before the operation, but I still experience some pain from time to time”.

Regarding the functional outcome, there were no statistically significant difference between the two groups in the early post-operative period in the OKS, suggesting no impact for GNRFA on the functional outcome which is compatible with the outcomes of a very recent studies done resembling our study [27–29].

The VAS scores also showed no statistically significant improvement in the TKA-GNRFA study group over the TKA study group, yet the VAS is a very subjective scoring system which is also hindered by the possibility if improvement in between the assigned time intervals of assessment specially between Six weeks and three to six months, since a single point improvement can be what made the patient feel better earlier, yet duo to its low value it cannot be interpreted statistically. And some studies showed improved pain scores as a reflection in decreased opioid consumption post-operative as a more objective assessment tool [30,31].

Patients suffering from residual pain showed in Table 4 reflect the true satisfaction rate of the patients, although 97% of the patients reported as satisfied, 14.7–20.59% were still having a complaint of minor to moderate pain. And this analysis showed that the higher impact of true satisfaction relies more on pain which will always have a reflection on the functional outcome.

## Limitations

The use of standardized perioperative and analgesia protocols diminishes the risk of bias but cannot eliminate the possibility of unconsciously affecting the surgeon’s surgical technique in a single blinded study. The use of a sham-controlled design couldn’t be fully utilized due to the nature of needle marks from the radiofrequency cannulas and put into consideration that it might also break the patients’ blinding. Although the outcome assessors were blinded the use of subjective measures like VAS can cause a limitation due to improvement between assessment intervals. The total postoperative opioid consumption can be subjective to pain tolerance of the patients, yet it’s strongly recommended as a secondary outcome and a reliable more objective pain measuring tool for further studies.

The use of a simple binary “yes or no” question for patients’ satisfaction reporting showed to be unreliable, as it fails to distinguish between the level of satisfaction clearly showed in the residual pain reports (14.7–20.59%) and we recommend the use of a more quantifiable scale or a specific satisfaction questionnaire.

A six months follow up is an early time point for final TKA outcome assessment and acknowledge that these results do not rule out a potential long-term benefit of GNRFA for the subset of patients who go on to develop chronic knee pain, hence longer follow up period is recommended for this matter.

## Conclusion

In our clinical trial we concluded that at 6 months postoperatively, combining GNRFA with TKA showed no significant advantage regarding the early postoperative pain scores and functional outcomes or patients’ satisfaction over TKA. Further randomized controlled trials with larger sample sizes are recommended to provide higher-level evidence and validate these findings.

## Data Availability

The datasets used and/or analyzed during the current study are available from the corresponding author on reasonable request. Assem M. M. Ahmed, Ahmed Sayed Kotb, Ahmed Mohamed Mohasseb contributed to the surgical procedures, clinical data collection and statistical analysis.

## Abbreviations

BMI: Body mass index
DAIR: Debridement, antibiotics, irrigation and implant retention
DM: Diabetes mellitus
DVT: Deep venous thrombosis
GNRFA: Genicular nerve radio frequency ablation
HTN: Hypertension
Hz: Hertz
LOCF: Last observation carried forward
MCID: Minimal clinically important difference
MSIS: Musculoskeletal infection society
NSAIDs: non-steroidal anti-inflammatory drugs
OKS: Oxford knee score
PCIA: Patient-controlled intravenous analgesia
PJI: Periprosthetic joint infection
RF: Radiofrequency
SPSS: Statistical package for social science
TKA: Total knee arthroplasty
TKA-GNRFA: Total knee arthroplasty combined with genicular nerve radiofrequency ablation
V: Volt
VAS: Visual analog scale

## Declarations

### Clinicial trial

Study adheres to CONSORT guidelines for clinical trials.
